# Smart prototype for an electronic color sensor device for visual simultaneous detection of macrofuran based on a coated paper strip

**DOI:** 10.1007/s00216-022-04374-z

**Published:** 2022-10-26

**Authors:** Sheta M. Sheta, Alaa S. Abdelelmoaty, Hassan M. Abu Hashish, Amira M. kamel, Mohkles M. Abd-Elzaher, Said M. El-Sheikh

**Affiliations:** 1 Department of Inorganic Chemistry, National Research Centre, Cairo, 12622 Egypt; 2 Mechanical Engineering Department, Engineering and Renewable Energy Research Institute, National Research Centre, Cairo, 12622 Egypt; 3 Department of Polymer and Pigments, National Research Center, Cairo, 12622 Egypt; 4 Department of Nanomaterials and Nanotechnology, Central Metallurgical R & D Institute, Cairo, 11421 Egypt

**Keywords:** Coated paper strip, Color sensor, Nano complex, Nitrofurantoin, Prototype device

## Abstract

Nowadays, in the clinical, pharmaceutical, and environmental sectors, the development of facile and sensitive analytical methods and/or innovative devices for the follow-up and detection of antibiotics and pharmaceutical formulations, in general, are urgently needed and still challenging. This work declared three vital applications for broad-spectrum nitrofurantoin (macrofuran) antibiotic detection and quantification: A colorimetric method, a coated paper strip-based nano-lanthanum complex prototype and fabrication of smart electronic color sensor device-based coated paper strips. The colorimetric method showed a significant response upon increasing the concentration of the nitrofurantoin in a range between (1.0–100.0 ng/mL) via a visual color change from orange-yellow to red colors degree with detection and quantification limits of 0.175 and 0.53 ng/mL, respectively, whereas the nano-lanthanum complex coated paper strip prototype showed qualitative on-site sensing for nitrofurantoin via naked eye color changes which can be detected anywhere. Moreover, a smart prototype for detecting macrofuran in the means of paper color change in the RGB color component extraction algorithm and the grayscale projection value processing algorithm was fabricated. The change in RGB color on the coated paper strip was detected using an electronic color sensor device. The developed colorimetric method, coated paper strip, and the electronic color sensor device prototype exhibited fast, simple, costless, and selective towards macrofuran over the competing analyzed. As well as, showed good applicability in the different real samples spiked with different concentrations of macrofuran.

**Graphical abstract:**

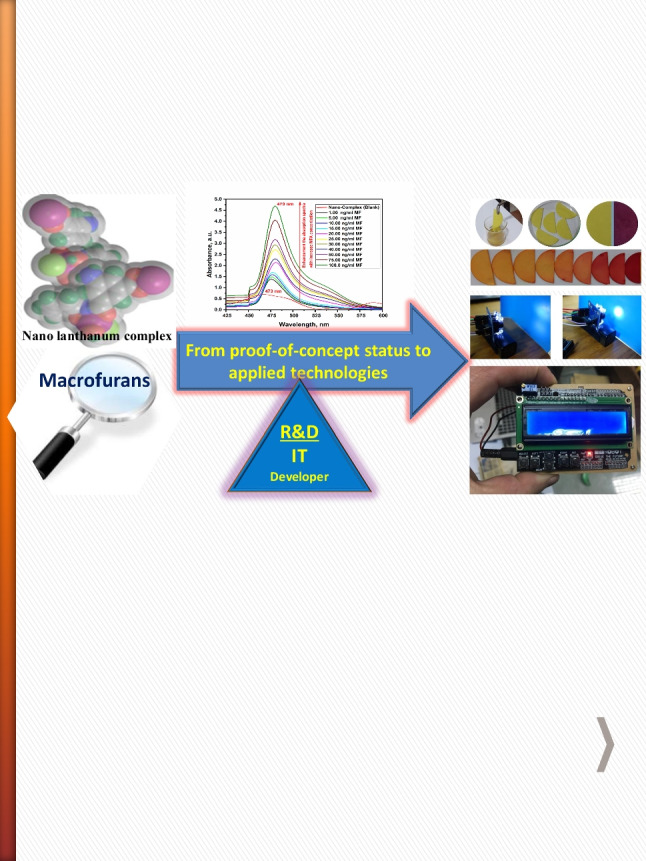

**Supplementary Information:**

The online version contains supplementary material available at 10.1007/s00216-022-04374-z.

## Introduction

Nowadays, many families of antibiotics and/or their metabolites have a potential cytotoxicity, human/animal health issues like hepatic or renal failure and sometimes [1–4]. In the clinical, pharmaceutical, and environmental sectors, the development of facile and sensitive analytical methods and innovative devices for the follow-up and detection of antibiotics and pharmaceutical formulations, in general, are urgently needed and still challenging [5–8]. On the other hand, point of care testing (POCT) is a terminology extensively used in the fields of healthcare, medical, pharmaceutical, environmental, and clinical applications [9–11]. POCT tools are used for early monitoring diseases, drug dose detection, pharmaceutical formulations screening, and as fast commercial diagnostic tools. Development of simple and fast detection tools and devices in terms of POCT is very important for drug dose control and screening and strategies of treatment without many steps of sample preparation, infra-construction instruments, and well-trained staff [12]. In addition, the requests for POCT for different fields like genetic testing, pharmacology, and healthcare are potential growth, leading to increasing the mission and put a future vision for continuous development and inducing innovative ideas in this important sector [13–16]**.**

In our previous work, [4] a novel nano-complex derived from a simple reaction of lanthanum chloride and phenylenediamine was synthesized and fully characterized using several spectroscopic tools. A physicochemical investigation was performed, and then the prepared nano-complex was used for the development of cost-effectiveness/fast/simple optical photoluminescence-based approach for macrofuran determination as a common antibiotic that is widely used for the treatment of the cases of urinary tract infections. The reported chemosensor showed high sensitivity/selectivity toward macrofuran and lower detection and quantification limits with a wide linear concentration range. Moreover, the applicability in different real biological samples and pharmaceutical formulations was investigated and studied [4].

In continuation of our previous work [4], development of facile and sensitive analytical methods and innovative devices to follow-up and detection of commonly used antibiotics and pharmaceutical formulations, in general, are urgently needed and still challenging. Herein, in this work using the previously prepared nano-lanthanum complex, three analytical approaches for the detection of macrofuran were declared; the first colorimetric method showed a significant response upon increasing the concentration of the macrofuran in a wide range with lower detection and quantification limits besides, significant visual color change from orange-red to red degree. The second declared a prototype of coated paper strip-based nano-lanthanum complex as a very costless, fast, and simple detection tool. The presented prototype showed qualitative on-site sensing for macrofuran via naked eye color changes which can be detected anywhere at any time from yellow to red degree. Finally, the fabrication of a prototype for color sensor via development of software application can easily quantitate different concentrations of the macrofuran as a function in the change in the RGB color map. The results obtained from the developed colorimetric method, coated paper strip, and the color sensor prototype device prove the high selectivity/selectivity, extra-fast detection, very low-cost analytical tools, and applicability in the different real samples and pharmaceutical formulations. These simple tools will help and facialized many sectors like clinical, pharmaceutical, and environmental as examples for the detection and screening like these types of commonly used antibiotics.

## Experimental

### Materials and instrumentation

The details of various materials used in preparation of the nano-lanthanum complex were described and reported by Sheta et al., [4]. In brief, 1, 2-phenylenediamine 99.5%, Lanthanum chloride; 99.99%, and nitrofurantoin; 99.9% were purchased from Sigma-Aldrich. The different antibiotics used as interfering analytes and pharmaceutical formulations were purchased from a local company. Whatman, grade I filter membrane, and other solvents and chemicals used in this study were of analytical reagent grade and were used as received.

The characterization and applications were performed using different instrumentation as follows: The FE-SEM images were recorded with a field emission scanning electron microscopy (JEOL-JSM-6510LV-Japan). The UV–vis measurements were obtained using V-770 UV–visible/NIR spectrophotometer (JASCO-USA). The measurements were performed in a quartz-cuvette of path length 1 cm, at room temperature, and the data were analyzed with Origin-8. The TCS3200 color sensor, Arduino Uno kit, and other electronics were purchased from the electronic market (Ram Company) with the following specifications: “Power = 2.7–5.5 V; Size = 1.12 × 1.12 inch; Interface = digital TTL; High-resolution in the conversion of light intensity into frequency; Programmable the change in color based on the output frequency; Directly communicated to microcontroller.”

## Procedures

### Synthesis of the nano-lanthanum complex

The nano-lanthanum complex was described and reported by Sheta et al. [4]. In brief, simple of 1.0 mmol LaCl_3._8H_2_O and 3.0 mmol phenylenediamine with stirring at room temperature for one day. Orange-red precipitate was formed, collected, filtered, washed, and dried.

### General procedure for macrofuran determination using the colorimetric method

A 1.0 mL of 1.0 mM of nano-lanthanum complex working solution was prepared in ethanol and subjected to UV–vis absorption as a blank sample in a quartz cuvette of path length 1 cm. Afterward, measured against 0.1 mL of freshly prepared concentrations of macrofuran standard. Under the optimization conditions, the absorption intensities were increased as the concentration macrofuran increase in a range between 1.0 and 100 ng/ml, and according to the linear-relationship equation: Y = a + bX “In which; Y, is the absorption intensities of the nano lanthanum complex; a and b are the intercept and slope of the linear-relationship, respectively; and X, is the nano lanthanum complex concentrations.” The LOD and LOQ have been estimated from the equations: “LOD = (SD/S)*3.3 & LOQ = (SD/S)*10 [17–19]. Where SD and S are the standard errors of absorption intensities; and the slope of the linear relationship, respectively.” Moreover, the nano-lanthanum complex solutions were subjected to absorption measurements against different similar antibiotics (as interfering analytes) in a separate cell and a mixture with macrofuran to investigate the selectivity.

### A prototype-based coated paper strip preparation and optimization

A portable prototype-based coated paper strip was prepared by using round Whatman sterile membrane filters 47 mm diameter. The papers were cut into two pieces equally and then soaked in a 100 mL beaker containing 100 mM of nano-lanthanum complex solution overnight. The coated paper strips were dried at 60 °C in the oven, and after drying became ready to use, the subsequently can be immersed in different concentrations of macrofuran as standard, pharmaceutical formulation, or spiked in different real samples.

### A color sensor prototype device fabrication and optimization

The TCS3200 color sensor was used and programmed as smart prototype for an electronic color sensor device-based coated paper strip to detect macrofuran in the means of paper color change in the RGB color component extraction algorithm and the grayscale projection value processing algorithm was fabricated.

### Applications in real samples and pharmaceutical formulations

The biological real samples (serum, plasma, and urine) are supplied from Family Medical Laboratory (FML), Ministry of Health, Egypt, and handled, pre-treated according to standard ethics and precautions guidelines and subsequently subject to measurements for UV–vis instrument and prototype-based coated paper strips as described in the above sections. Additionally, the pharmaceutical formulations containing nitrofurantoin (macrofuran 100 mg capsules) are supplied from local drug stores. The formulation stock solution preparation is performed as described according to Sheta et al. [4] and subject to different measurements as described in the above.

## Results and discussion

### Macrofuran quantitative determination using the colorimetric method

**Fig. 1:**
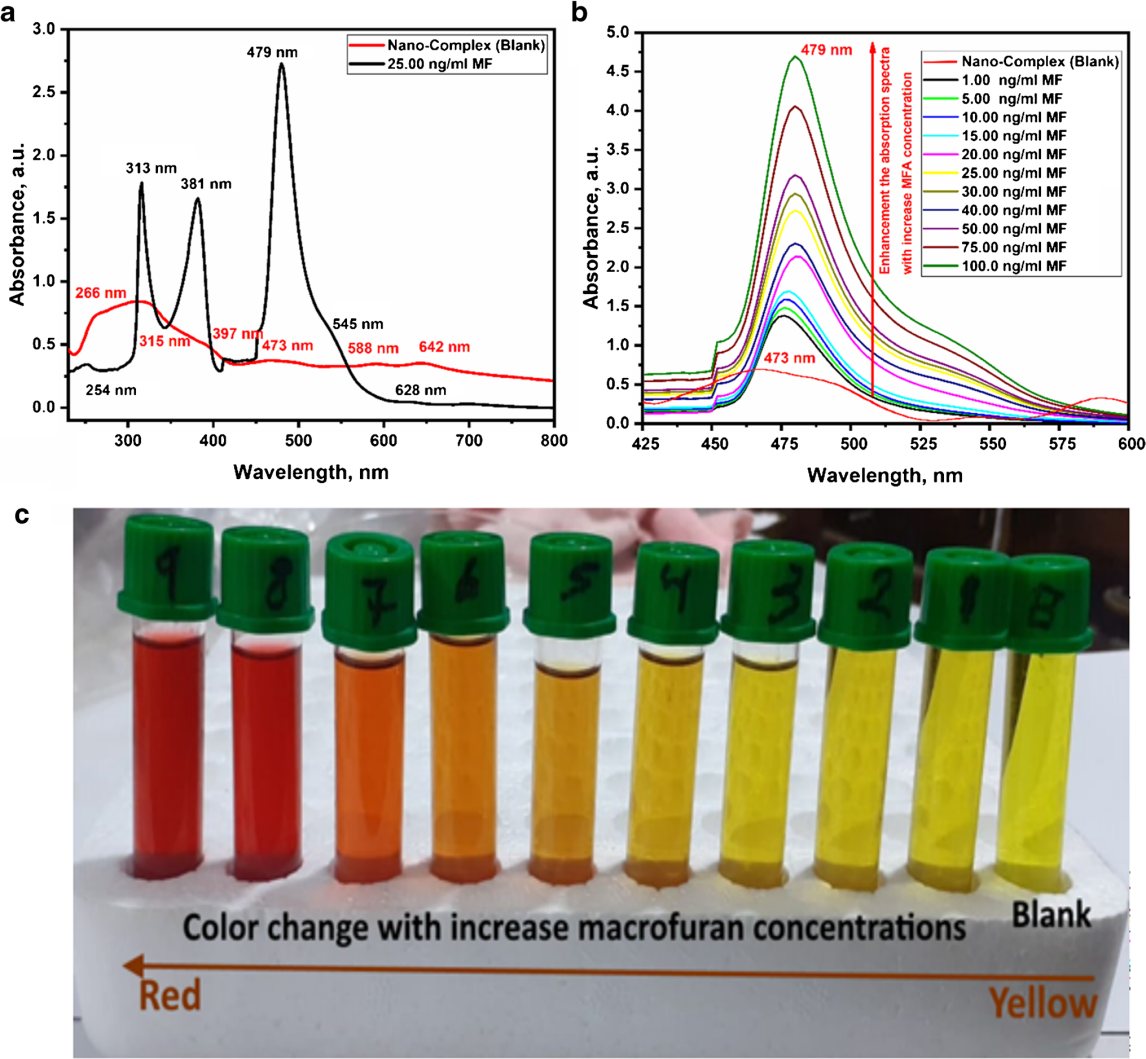
**a** The UV-absorption spectrum of the nano-La complex (red color) and in with addition of 25 ng/mL macrofuran (black color). **b** The UV-absorption spectrum of the nano-La complex against different concentrations of macrofuran at 479 nm. **c** A smartphone photography image for color change of the nano-La complex with increasing the concentrations of macrofuran

From our previous study of the UV-absorption spectrum of the nanο-La complex presented in [4], it showed six absorption peaks at “266, 315, 397, 473, 588 and 642 nm due to the intra-ligand π- π*, n-π*, and LMCT” [4]. However, the 25 ng/mL macrofuran@nano-La complex exhibited six UV–vis peaks at 254, 313, 381, 479, 545, and 628 nm as represented in Fig. 1a. Comparing the absorption spectra of nano-La complex with the macrofuran@nano-La-complex, we noted a blueshift for the all peaks except at the peak at 480 nm, a red-shift is observed (from 266 to 254 nm; 315 to 313 nm; 397 to 381 nm; 588 to 545 nm; 642 to 628 nm “blueshift”; and from 473 to 479 nm “red-shift”). In addition to, significantly increasing the intensities of the peaks at 316, 381 and 479 nm (sharp peaks).

The nano-La complex was tested as a colοrimetric chemosensor for the macrofuran detection and quantification. The UV-absorption spectrum of the nanο-La-complex was investigated against different concentrations of macrofuran, and the results were presented in Fig. S1. As revealed in Fig. S1, we can be observed a significant increase of the absorption spectra peaks at 313, 381, and 479 nm.

Focusing on the sharp peak at 479 nm showed a red-shift with about 6 nm with a color change from orange-yellow to red color (Fig. 1b). Additionally, by increasing the macrofuran concentration from 1.0 to 100.0 ng/mL the absorption peak intensities increased gradually. Moreover, the colors of the nano-La complex solutions were converted from orange-yellow to red color shown in Fig. 1c. Accordingly, the nano-La complex could be used as a naked eye indicator for macrofuran and colorimetric chemosensor.

**Fig. 2:**
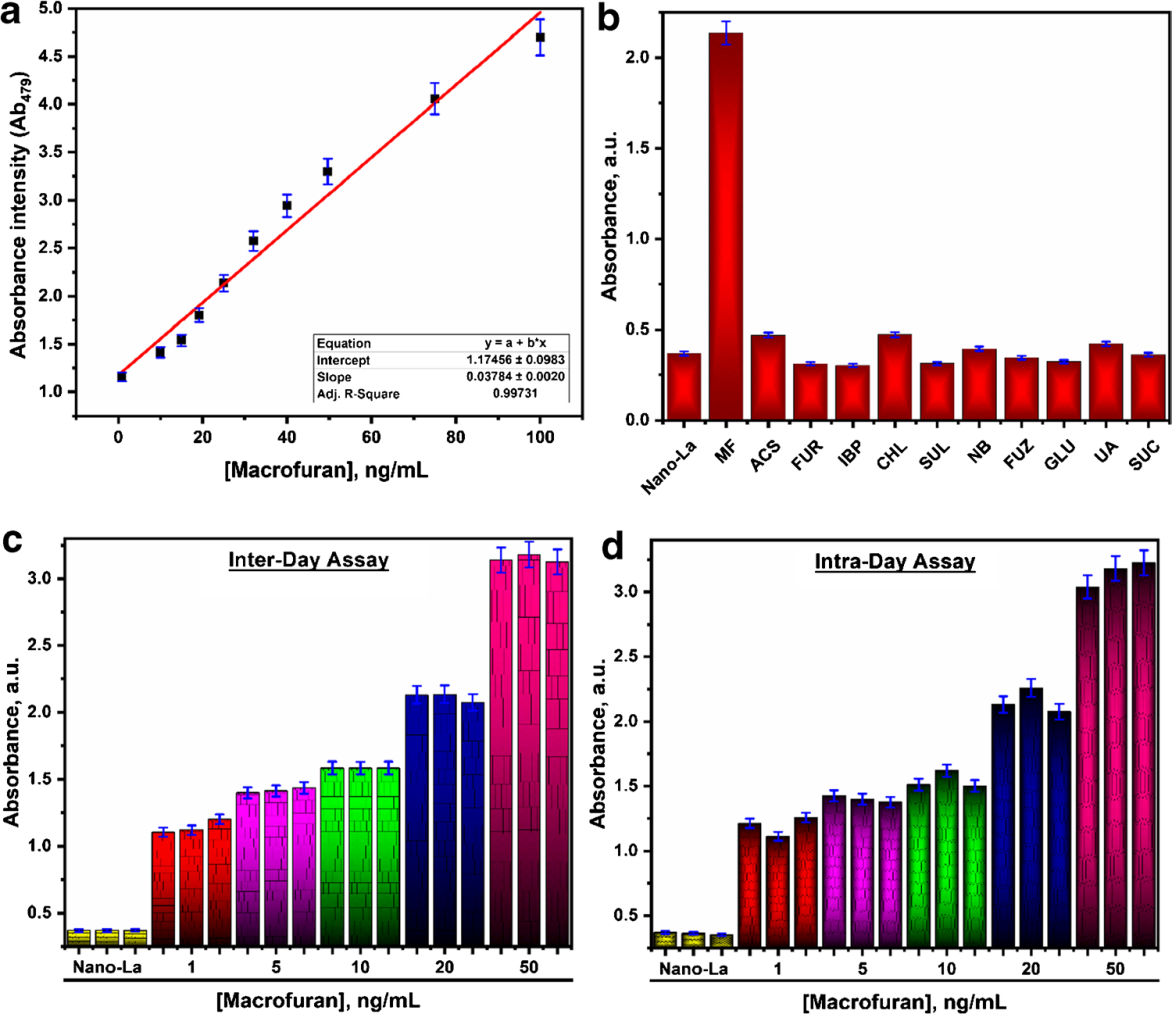
**a** A linear relationship (calibration graph) between the nano-La complex absorbance intensities and different macrofuran concentrations. **b** A histogram of evaluation of the absorption intensity of the nano-La complex towards the macrofuran against different types of interfering analytes. **c** A histogram of evaluation of inter-day accuracy and precision for the colorimetric method. **d** A histogram of evaluation of intra-day accuracy, and precision for the colorimetric method

Under the optimum conditions, as presented in Fig. 2a, a linear relationship was achieved between the nano-La complex absorbance intensities and different macrofuran concentrations. On the absorption peak intensities at 479 nm (Ab_479_), the calibration curves of the proposed colοrimetric method showed stability response in a wide concentration in a range of 1.0–100.0 ng/mL, and the fitted Eqs. (1) can express as:

1 $$\mathrm{Absorbance intensity}\left({\mathrm{Ab}}_{479}\right)=1.175+0.038\;\left[\mathrm{Macrofuran}\right] \mathrm{with }{r}^{2}=0.9973,$$

The colorimetric method-based on nano-La complex exhibited excellent sensitivity towards macrofuran with a detection limit (LOD) of 0.175 ng/mL and quantification limit (LOQ) of 0.53 ng/mL; the summarized of best optimized conditions for the proposed colοrimetric method and regression parameters was presented in Table 1. Comparison of the present method with previously published work [1–4, 20–26] was presented in Table S1.

**Table 1:** Sensitivity and regression parameters for colorimetric method

Parameter	Method
Absorbance, nm	479
Limit of detection (LOD), ng/mL	0.175
Limit of quantification (LOQ), ng/mL	0.53
Regression equation	(Y = a + bX)*
Intercept (a)	1.17456
Slope (b)	0.03784
Standard deviation	0.002
Correlation coefficient (*r*^2^)	0.9973

^*^Y is the absorbance intensities of nano-La complex; X is the concentration of macrofuran in ng/mL; a is intercept; b is slope

The effects of pH and temperature on the absorption peak intensities at 479 nm (Ab_479_) of the present colorimetric method at different macrofuran concentrations were investigated. The results revealed that changing the pH from about 3.5 to 10.5 did not seem to cause any significant changes in the absorbance intensities. However, the change in environmental temperature from 18 to 35 °C also did not seem to cause any significant changes in the absorbance intensities. The influence of the solvents on the absorption peak intensities at 479 nm (Ab_479_) of the present colorimetric method at concentrations also was investigated and studied. The results revealed the high absorption intensity of the system in ethanol and then water because these solvents stabilize the excited state of the sensor. However, with other solvents like acetonitrile, DMF, and DMSO, the absorption intensity of the sensor is slightly decreased and/or a blue shift due to the destabilization of the excited state by these solvents.

The evaluation of the potential selectivity and specificity of nano-La complex towards macrofuran based on the current colorimetric method was performed according to a similar study in our previous work [4]. However, herein, the absorption spectra of nano-La complex (1.0 mM) were examined against the interfering substances mention before like “Acetylsalicylic acid (ACS), Chloramphenicol (CHL), Furosemide (FUR), Furazolidone (FUZ), Glucose (GLU), Ibuprofen (IBP), Nitrobenzene (NB), Sucrose (SUC), Sulfafurazole (SUL), and Uric acid (UA)”) at the concentration level of 20.0 ng/mL for the macrofuran and interfering substances and presented in Fig. 2b. As shown in the presented histogram, the results of the Abs_479_ intensities were extremely enhanced with macrofuran, without changes in the case of the other interfering matrix. The obtained results were in excellent agreement with the obtained ones in our previous study [4], which revealed that the nano-La complex is extremely selective for macrofuran.

The reducibility and repeatability of the presented method were evaluated via study the inter- and intra-day accuracy and precision. This study was investigated at 5 levels of macrofuran concentrations (1.0, 5.0, 10.0, 20.0, and 50.0 ng/mL), and each reading was replicated 3 times. The histograms of Abs479 intensities for inter-/intra-days were presented in Fig. 2c and d, respectively. From the histogram, results prove the accuracy, precision, reducibility, and repeatability of the present work.

The applicability of the present colorimetric method for determination of macrofuran in different real samples (serum\plasma\urine) as well as in pharmaceutical formulations including the recoveries study was investigated. The investigation was carried out via spiking method at three concentration levels of macrofuran (1.0, 10.0, and 50.0 ng/mL) at different serum\plasma\urine samples and then evaluated the recoveries percent, and the summarized results were presented in Table S2. However, the pharmaceutical formulations of macrofuran “Capsules 100 mg” were examined at the same above levels (1.0, 10.0, and 50.0 ng/mL) of concentration and the data was presented in Table S2. From the table data, the average recoveries percent were about 97.44, 97.88, 100.67, and 98.30% for serum, plasma, urine, and pharmaceutical formulation samples, respectively. These results prove that the proposed method is applicable, sensitive, and effective, for quantification of macrofuran different pharmaceutical formulation and real samples; and this method will be a future promising analytical tool for simple and fast macrofuran detection and quantification.

### Macrofuran qualitative detection using prototype-based coated paper strip

**Fig. 3:**
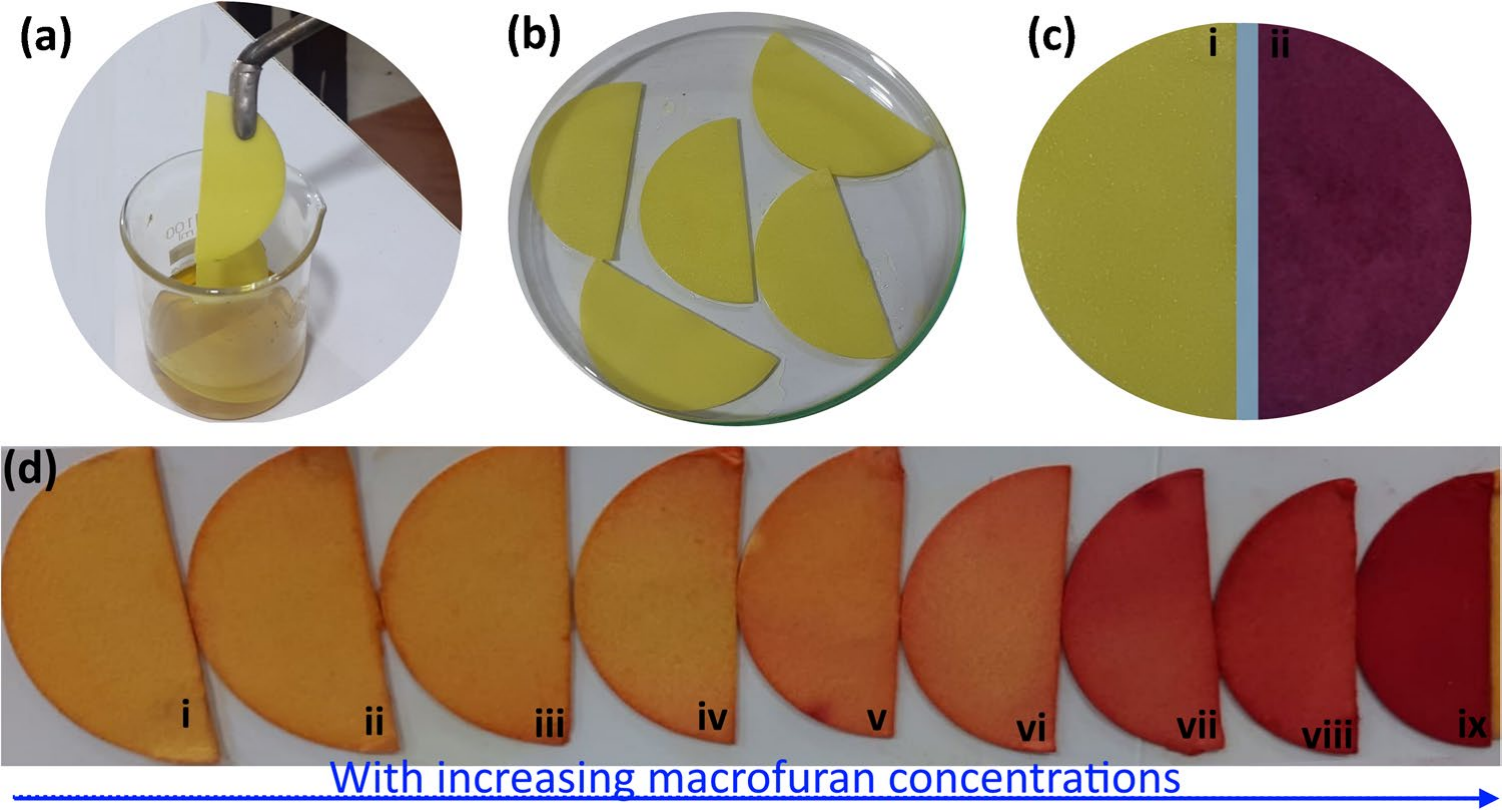
A smartphone photography images for prototype-based coated paper strip preparation and optimization: **a** Soaking step in beaker contain nano-lanthanum complex solution. **b** Drying step and ready to used coated paper strips. **c** A coated paper strip optimization before “blank” [i] and after immersed in 100 ng/mL of macrofuran [ii]. **d** A change in the degree of color based on the change of concentrations of macrofuran [i, 1.0 ng/mL; ii, 5.0 ng/mL; iii, 10.0 ng/mL; iv, 20.0 ng/mL; v, 30.0 ng/mL; vi, 40.0 ng/mL; vii, 50.0 ng/mL; viii, 75.0 ng/mL; and ix, 100.0 ng/mL]

As clarified in the experimental section, the prototype-based coated paper strip was prepared by soaking the Whatman sterile membrane in a beaker containing 100 mM of nano-lanthanum complex solution overnight as shown in Fig. 3a. After that, the coated paper strips were dried at 60 °C in the oven in a petri dish as shown in Fig. 3b, and then after drying, the prototype-based coated paper strip became ready to use. By immersing this strip in 100 ng/mL of macrofuran, the color changes during 30 s from yellow to red color as shown in Fig. 3c; these strips can be used as naked eye detection of macrofuran (as macrofuran indicator). Moreover, by increasing the concentrations of macrofuran the color degree in transfer from yellow color to red degree to deep red colors as shown in Fig. 3d.

The validation and optimization of the present prototype-based coated paper strips for detection of macrofuran were investigated via study of the applicability in real samples as well as in pharmaceutical formulation, beside the interfering and selectivity evaluation, with a similar study mentioned before in the above colorimetric method. The summarized results of this investigation showed that the color of coated paper strips changed directly based on the concentration of macrofuran. Moreover, the coated paper strips showed high sensitivity toward macrofuran via color change to red, whereas nothing occurs with other interfering.

### Electronic color sensor device prototype fabrication and optimization for macrofuran quantitative detection based coated paper strip

As presented in Fig. 3d, the red color intensities are directly proportional to the macrofuran concentration. So, we can benefit from the change in color of coated paper strips as a function in macrofuran concentration change via fabrication of an electronic color sensor device prototype for quantitative detection of macrofuran. The sensing fabrication strategy with programing details are discussed as follows:

#### TCS3200 color sensor specifications and connection process

**Fig. 4:**
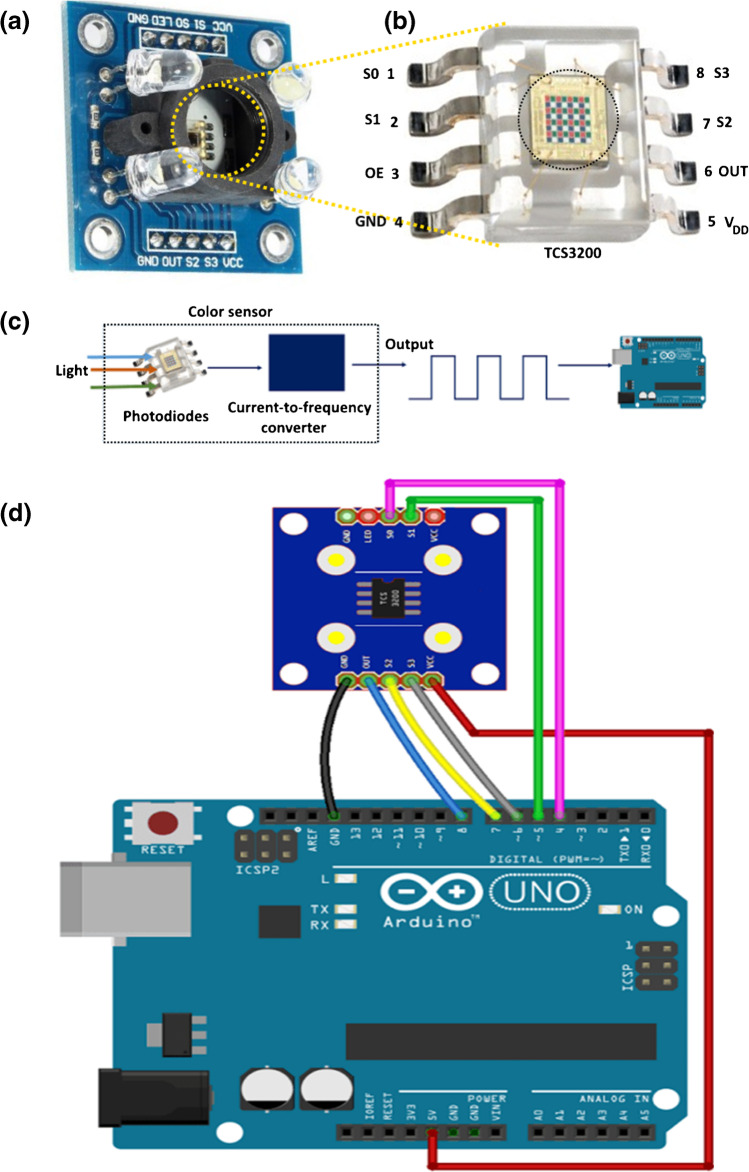
**a** The TCS3200 color sensor, **b** TCS3200 RGB sensor chip, **c** the photodiode (color sensor) connected to Arduino, and **d** wiring the TCSP3200 sensor to your Arduino; simply follow the next schematic diagram

The TCS3200 color sensor as shown in Fig. 4a generally uses a RGB sensor chip for the color detect process [27]. The details of the color sensor component and mechanism of work were presented in the supporting data file and according to Fig. 4b and c. Moreover, the details of control pin connection data are summarized in TCS3200 sensor data sheet (Table S3). Finally, the TCSP3200 sensor wiring connection to Arduino Uno according to the schematic diagram (Fig. 4d).

#### Preparing the sensor code for Arduino and data interpretations

Preparation the sensor code programming for Arduino and data interpretations can simplified in the following two stages:

##### Reading the output frequency

Reading color and displaying the concentrations according to output frequency on the monitor. The frequency values were written down when you place different colors in the color sensor. Overall, this stage carried out in the following steps:

- Uploading the code to the Arduino-board according to Scheme S1.
- Opening the serial-monitor at 9600 baud-rate.
- To select the best distance for the sensor, in front of the sensor put a blue color object at dissimilar distances as shown in Fig. S2.
- Optimizing the best distance by saving the two measurements: when the object is placed far and near from the sensor, respectively.
- The values were checked on the display. The frequency of blue color (B) compared to the reading frequency of red color (R) and green color (G) should be the lower value according to Scheme S2.
- At optimum blue object position at the front of the sensor, the values of the frequency of blue color (B) oscillate between 59 and 223 (see the values highlighted in Scheme S2). In our case, the frequency values (59 and 223) cannot use in code preparation. It should measure the colors for the present specific object with our own color sensor followed by saving the obtained upper and bottom frequency limits for the blue color.
- Repeat the above step with red and green objects and save also the frequency limits (upper and bottom) for each color.

##### Characterization the different colors

The difference between colors was recorded in the program. The frequency values were calibrated according to the concentration of color previously on the program code; the sensor can distinguish between different concentrations related to RGB. Overall, the values frequency maps to RGB between 0 and 255. In the former step, the corresponding frequency at blue maximum was 59 and at higher distance was 223. So, the frequency values of 59 and 223 are corresponding to 255 and 0 in the RGB, respectively. Subsequently, according to Scheme S3 at the Arduino map, () function are replaced with your own values. Finally, to obtain the concentration through the distinguish between the change in colors by reading of the red, green, and blue values.

Now, the program compares the read value to the storge value in Table 2, and if the value is in the range of 80% of any value of the table, the program given concentration at these values. The obtained data according to macrofuran quantitative detection-based coated paper strip using the electronic color sensor device prototype is summarized in Table 2. A smartphone photo for the primary version prototype of the electronic color sensor was present in Fig. S3.

**Table 2:**
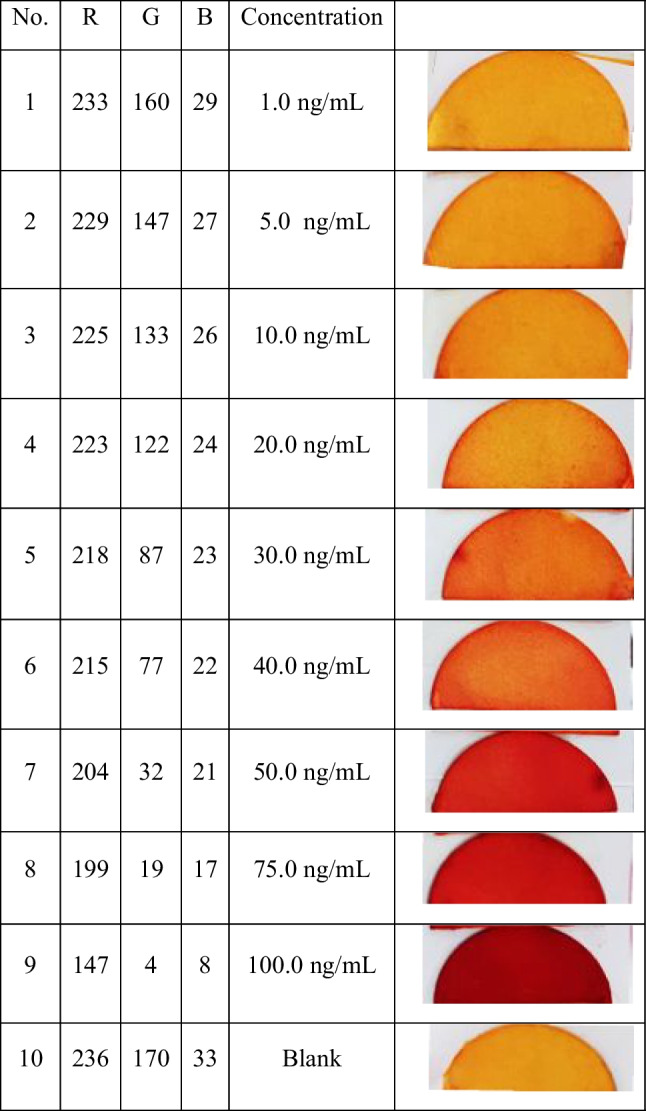
Distinguish between different colors

Finally, all of the above results concluded that the suggested prototype device will be promising, efficient, sensitive, selective, accurate, precise, lower cost, friendly, easy test, working with different real samples (serum\plasma\urine) as well in pharmaceutical formulation for monitoring and quantification of macrofuran.

## Conclusion

This work presents three critical applications, the first a colorimetric method-based nano-lanthanum complex for determination of macrofuran concentration in different real samples (serum/plasma/urine) as well in pharmaceutical formulation. In the colorimetric method, results showed a significant visual color change from orange-red to red degree which can fast and easily detect of the macrofuran at once with naked eye. Moreover, the present method showed a significant response upon increasing the concentration of the macrofuran in a wide range with lower detection and quantification limits. The statistical assessment of the presented method is evaluated and was fit for purpose and introduces a lot of figures of analytical merits. The second application was developing a prototype-based coated paper strip for qualitative detection macrofuran via naked eye color change. Finally, fabrication and optimization of an electronic color sensor device prototype for macrofuran quantitative detection-based coated paper strip. The summarized data revealed that the three applications showed a fast, simpler, less cost, extra-sensitive, and ultra-selective, no need for complicated technology, depend on color change, home users test, applicable for different types of biological samples (serum/plasma/urine), and friendly. Moreover, these applications will be promising future crucial tools for monitoring and/or quantifying one of the harmful antibiotics the world has widely used and causing many issues on humans, animals, and the environment in general.

## Supplementary Information

Below is the link to the electronic supplementary material.

Supplementary file1 (DOCX 932 KB)
