# Supplementary material for: Smart prototype for an electronic color sensor device for visual simultaneous detection of macrofuran based on a coated paper strip
Source: Anal Bioanal Chem. 2022 Oct 26;414(29-30):8379–88. doi: 10.1007/s00216-022-04374-z (PMC9712405; doi:10.1007/s00216-022-04374-z)
Supplement: Supplementary file 1 — Supplementary file1 (DOCX 932 KB) [file 216_2022_4374_MOESM1_ESM.docx]

**Supporting Information**

*For*

**Smart prototype for an electronic color sensor device for visual simultaneous detection of macrofuran based on a coated paper strip**

**Sheta M. Sheta^1, *^, Alaa S. Abdelelmoaty^1^, Hassan M. Abu Hashish^2^, Amira M. kamel^3^, Mohkles M. Abd-Elzaher^1^, Said M. El-Sheikh^4,*^**

*^1^ Department of Inorganic Chemistry, National Research Centre, Cairo, 12622, Egypt.*

*^2^Mechanical Engineering Department, Engineering and Renewable Energy Research Institute, National Research Centre, Cairo, 12622, Egypt.*

*^3^  Department of Polymer and Pigments, National Research Center, Cairo, 12622, Egypt.*

*^4^ Department of Nanomaterials and Nanotechnology, Central Metallurgical R & D Institute, Cairo, 11421, Egypt.*

**Contents**

**The TCS3200 cοlor sensοr specifications and connection process**

**Scheme S1:** The sensor Code written on Arduino board

**Scheme S2:** The readings of color frequency scheme

**Scheme S3:** Final project to view colors value

**Fig. S1.** The absorption spectra response for behavior of the nano-La-complex towards different concentrations of macrofuran.

**Fig. S2.** Optimization of the sensor and object position.

**Fig. S3.** Smartphone photo for the primary prototype version of an electronic color sensor.

**Table S1.** Comparison between the colorimetric method-based nano-lanthanum complex and some existing methods for the determination of macrofuran.

**Table S2.** Determination of macrofuran in different real samples and pharmaceutical formulation using nano-lanthanum complex-based the colorimetric method.

**Table S3.** The TCS3200 Sensor data sheet

****Corresponding author:***

*Sheta M. Sheta; E-mail: dr.sheta.nrc@gmail.com,; Tel.: +20 1009697356; Fax: +2-02-33370931.*

**The TCS3200 cοlor sensοr** **specifications and connection process.**

The TCS3200 cοlor sensοr as shοwn in (Fig. 4 a), generally uses a RGB sensοr chip for the cοlοr detect prοcess. It contains four white LEDs that light up the object in front of it (27). TCS3200 sensor work according to the follow mechanism: the TCS3200 has an array of photodiodes with 4 different filters. A photodiode is simply a semiconductor device that converts light into current. The sensor has: 16 photodiodes with red filter – sensitive to red wavelength, 16 photodiodes with green filter – sensitive to green wavelength, 16 photodiodes with blue filter – sensitive to blue wavelength, and finally, 16 photodiodes without filter (Fig. 4 b). If you take a closer look at the TCS3200 chip you can see the different filters (Fig. 4 b).

By selectively choosing the photodiode filter’s readings, you’re able to detect the intensity of the different colors. The sensor has a current-to-frequency converter that converts the photodiodes’ readings into a square wave with a frequency that is proportional to the light intensity of the chosen color. This frequency is then, read by the Arduino as shown in (Fig. 4 c). The sensor pinout and connection according to pin name as showed in (Fig. 4 b) is summarized in TCS3200 sensor data sheet (Table S2). To select the color read by the photodiode, you use the control pins S2 and S3. As the photodiodes are connected in parallel, setting the S2 and S3 LOW and HIGH in different combinations allows you to select different photodidodes as presented in data sheet (Table S2). For frequency scaling: Pins S0 and S1 are used for scaling the output frequency. It can be scaled to the following preset values: 100%, 20% or 2%. Scaling the output frequency is useful to optimize the sensor readings for various frequency counters or microcontrollers as presented in data sheet (Table S2). For the Arduino, it is common to use a frequency scaling of 20%. So, you set the S0 pin to HIGH and the S1 pin to LOW.

The TCSP3200 sensor was wiring to Arduino Uno as the following schematic diagram (Fig. 10d). the TCSP3200 connected to the Arduino as shown in (Fig. 4 d) as following: (S0: digital pin 4, S1: digital pin 5, VCC: 5V, S3: digital pin 6, S4: digital pin 7, OUT: digital pin 8) (Fig. 4 d).

/*** Smart prototype for visual simultaneous detection of macrofurans ***/

// Sensor pins number on Arduino

#define S0 1

#define S1 2

#define S2 3

#define S3 4

#define sensorOut 5

// frequency range

int redFrequency = 0;

int greenFrequency = 0;

int blueFrequency = 0;

void setup() {

// sensor outputs setting

pinMode(S0, OUTPUT);

pinMode(S1, OUTPUT);

pinMode(S2, OUTPUT);

pinMode(S3, OUTPUT);

// sensor input from output setting

pinMode(sensorOut, INPUT);

// frequency calibration at 20%

digitalWrite(S0,HIGH);

digitalWrite(S1,LOW);

// serial communication value

Serial.begin(9600);

}

void loop() {

// R filter read

digitalWrite(S2,LOW);

digitalWrite(S3,LOW);

// frequency output

redFrequency = pulseIn(sensorOut, LOW);

// Printing value ofR

Serial.print("R = ");

Serial.print(redFrequency);

delay(100);

// G filter read

digitalWrite(S2,HIGH);

digitalWrite(S3,HIGH);

// frequency output

greenFrequency = pulseIn(sensorOut, LOW);

// // Printing value of G

Serial.print(" G = ");

Serial.print(greenFrequency);

delay(100);

// B filter read

digitalWrite(S2,LOW);

digitalWrite(S3,HIGH);

// frequency output

blueFrequency = pulseIn(sensorOut, LOW);

// // Printing value of B

Serial.print(" B = ");

Serial.println(blueFrequency);

delay(100);

}

**Scheme S1:** The sensor Code written on Arduino board


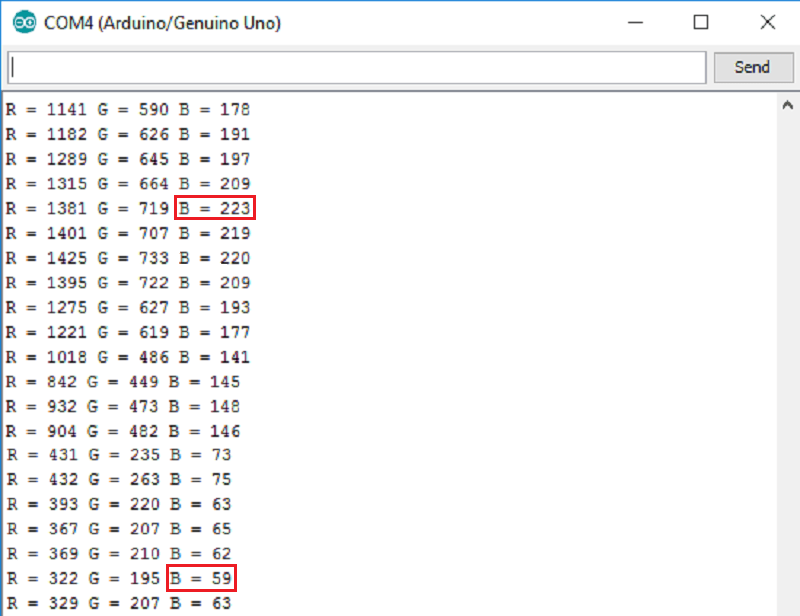


**Scheme S2:** The readings of color frequency scheme

/*** Smart prototype for visual simultaneous detection of macrofurans ***/

// Sensor pins number on Arduino

#define S0 1

#define S1 2

#define S2 3

#define S3 4

#define sensorOut 5

// frequency range

int redFrequency = 0;

int greenFrequency = 0;

int blueFrequency = 0;

// the color Stores of green, blue and red

int redColor = 0;

int greenColor = 0;

int blueColor = 0;

void setup() {

// sensor outputs setting

pinMode(S0, OUTPUT);

pinMode(S1, OUTPUT);

pinMode(S2, OUTPUT);

pinMode(S3, OUTPUT);

// sensor input from output setting

pinMode(sensorOut, INPUT);

// frequency calibration at 20%

digitalWrite(S0,HIGH);

digitalWrite(S1,LOW);

// serial communication value

Serial.begin(9600);

}

void loop() {

// R filter read

digitalWrite(S2,LOW);

digitalWrite(S3,LOW);

// frequency output

redFrequency = pulseIn(sensorOut, LOW);

// the Red frequency value range 0 : 255

// newvalues replaced to XX . :

// for example redColor = map(redFrequency, 70, 120, 255,0);

redColor = map(redFrequency, XX, XX, 255,0);

//

// Printing value of R

Serial.print("R = ");

Serial.print(redColor);

delay(100);

// G filter read digitalWrite(S2,HIGH);

digitalWrite(S3,HIGH);

// frequency output

greenFrequency = pulseIn(sensorOut, LOW);

// the GREEN frequency value range 0 : 255

// newvalues replaced to XX . :

// for example greenColor = map(greenFrequency, 100, 199, 255, 0);

greenColor = map(greenFrequency, XX, XX, 255, 0);

// // Printing value of G

Serial.print(" G = ");

Serial.print(greenColor);

delay(100);

// B filter read

digitalWrite(S2,LOW);

digitalWrite(S3,HIGH);

// frequency output

blueFrequency = pulseIn(sensorOut, LOW);

// the BLUE frequency value range 0 : 255

// newvalues replaced to XX . :

// for example blueColor = map(blueFrequency, 38, 84, 255, 0);

blueColor = map(blueFrequency, XX, XX, 255, 0);

// // Printing value of B

Serial.print(" B = ");

Serial.print(blueColor);

delay(100);

// color Check and printout

// serial monitor message

if(redColor > greenColor && redColor > blueColor){

Serial.println(" - RED detected!");

}

if(greenColor > redColor && greenColor > blueColor){

Serial.println(" - GREEN detected!");

}

if(blueColor > redColor && blueColor > greenColor){

Serial.println(" - BLUE detected!");

}

}

**Scheme S3:** Final project to view colors value


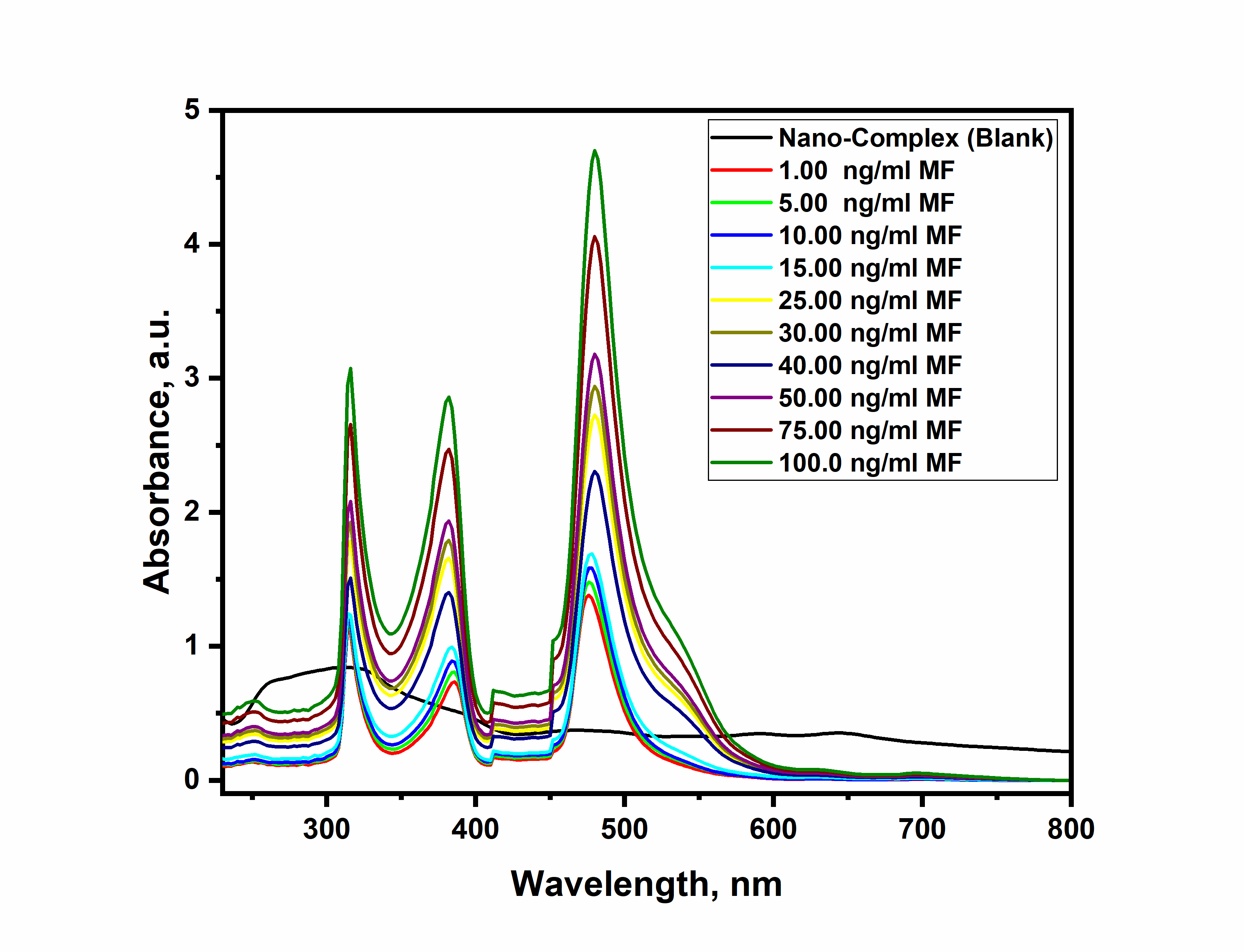


**Fig. S1.** The absorption spectra response for behavior of the nano-La-complex towards different concentrations of macrofuran.


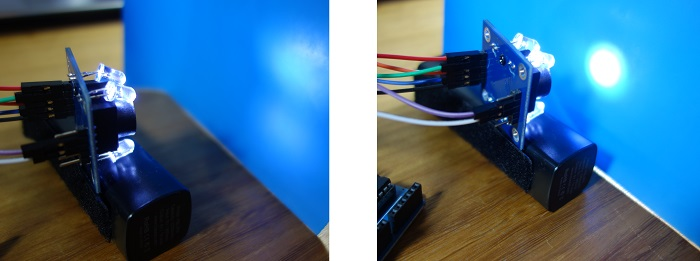


**Fig. S2.** Optimization of the sensor and object position.


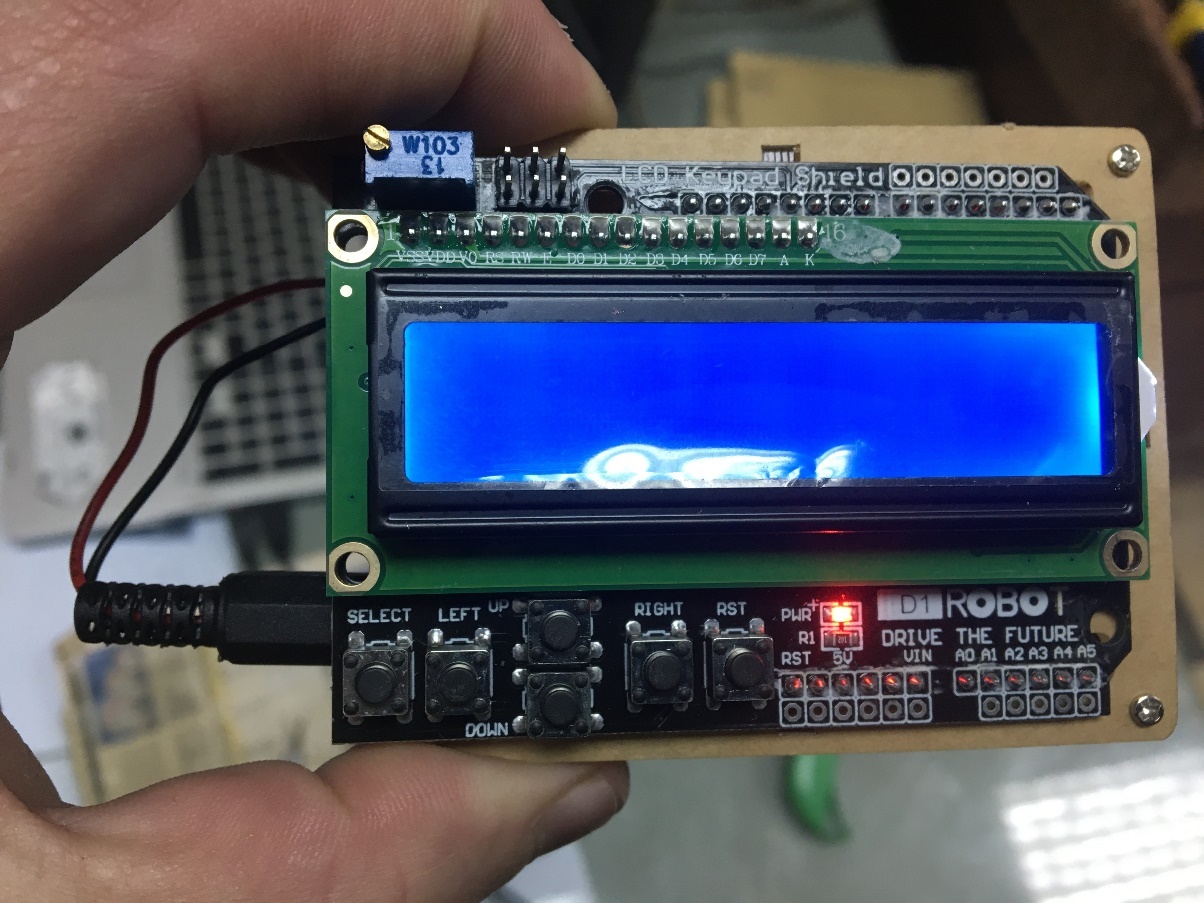


**Fig. S3.** Smartphone photo for the primary prototype version of an electronic color sensor.

**Table S1.** Comparison between the colorimetric method-based nano-lanthanum complex and some existing methods for the determination of macrofuran.

| **Ref** | **LOD** | **Linear detection range** | **Type of samples** | **Method** |
| --- | --- | --- | --- | --- |
| (1) | 1.14 nM | 0.005 – 100.0 µM | Pork liver samples | Electrochemical sensor based on reduced graphene oxide/Fe3O4 |
| (2) | 0.15 nM | 1.0 - 1000 nM | Feed samples | Voltammetric Method |
| (3) | 72.00 nM | 0.01 – 144.0 µM | Nile water samples | Electrochemical sensor based on lanthanum molybdate nanospheres |
| (20) | 0.30 nM | 0.001 - 0.05 µM;  0.100 - 1.0 µM | Animal serum samples | Electrochemical sensor based on molecularly imprinted copolymer |
| (21) | 8.00 nM | 0.085 – 1.01 μM | --- | Spectrofluorimetric Method |
| (22) | 30.00 nM | 0.05 – 4.00 μM | Lake water samples | Label-free photoluminescence assay |
| (23) | 8.03 nM;  20.28 nM | 0.021 – 1.27 nM | Pharmaceutical formulations | Flow injection-spectrophotometry |
| (24) | 0.60 µM | 10.0 – 100.0 μM | Feed samples | Electrogenerated chemiluminescence |
| (25) | 0.211 µM | 2.11 ‐ 42.25 µM | Rain water - mouthwash | Surface enhanced Raman spectroscopy |
| (26) | 0.114 nM;  0.19 nM | 0.211 - 5.28 nM;  0.017- 0.85 nM | Plasma-Urine- | UHPLC-DAD assay |
| (4) | 0.025 ng/mL  ~ (0.10 nM) | 0.02-30.0 ng/mL  ~(0.08-126.8 nM) | Pure form- Serum-Plasma-Urine- Pharmaceutical formulations | Nano-lanthanum complex chemosensor |
| The present work | 0.175 ng/mL | 1.0 – 100.0 ng/mL |  | Colorimetric method-based nano-lanthanum complex |
|  | - | - |  | Coated paper strips |
|  | - | - |  | Electronic color sensor device |

**Table S2.** Determination of macrofuran in different real samples and pharmaceutical formulation using nano-lanthanum complex-based the colorimetric method

| **Sample** | **Spiked/Conc macrofuran (ng/mL)** | **Found (ng/mL)** | | | **Average Found (ng/mL)** | **Recovery %** |
| --- | --- | --- | --- | --- | --- | --- |
| **Serum** | 1.0 | 0.935 | 0.969 | 1.058 | 0.987 | 98.74 |
|  | 10.0 | 9.63 | 9.515 | 9.685 | 9.61 | 96.1 |
|  | 50.0 | 47.41 | 47.73 | 51.09 | 48.74 | 97.48 |
| **Plasma** | 1.0 | 0.976 | 0.969 | 0.963 | 0.969 | 96.89 |
|  | 10.0 | 9.89 | 9.883 | 9.871 | 9.881 | 98.81 |
|  | 50.0 | 49.38 | 49.41 | 48.1 | 48.96 | 97.92 |
| **Urine** | 1.0 | 1.108 | 0.972 | 1.029 | 1.036 | 103.6 |
|  | 10.0 | 9.787 | 10.31 | 9.82 | 9.972 | 99.72 |
|  | 50.0 | 48.43 | 49.76 | 49.81 | 49.33 | 98.67 |
| **Pharmaceutical formulation** | 1.0 | 0.949 | 0.958 | 1.018 | 0.975 | 97.49 |
|  | 10.0 | 9.72 | 9.68 | 10.34 | 9.915 | 99.15 |
|  | 50.0 | 51.74 | 47.81 | 47.84 | 49.13 | 98.26 |

**Table S3.** The TCS3200 Sensor data sheet

**
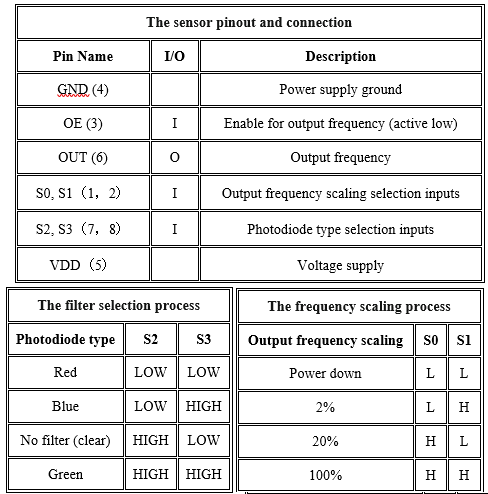
**
